# Tourist Trampling on a Peripheral Plant Population Restricted to an Urban Natural Area in the Capo Sant’Elia Promontory (Sardinia, W-Mediterranean Basin)

**DOI:** 10.3390/plants13060881

**Published:** 2024-03-19

**Authors:** Donatella Cogoni, Giulia Calderisi, Daniele Collu, Giuseppe Fenu

**Affiliations:** Department of Life and Environmental Sciences, University of Cagliari, Via S. Ignazio da Laconi 13, I-09123 Cagliari, Italy; d.cogoni@unica.it (D.C.); giulia.calderisi@unica.it (G.C.); danielecollu97@gmail.com (D.C.)

**Keywords:** human trampling effects, *Globularia alypum* L., isolated plant population, mediterranean flora, natural urban area, plant conservation, Sardinia

## Abstract

Urban natural areas provide important ecological services such as biodiversity conservation, as well as opportunities for people to connect with nature and preserve cultural heritage. However, the increasing demand for access to natural areas and the expansion of human recreational activities, such as hiking and biking, pose threats to these ecosystems, especially for animal and plant species, finally resulting in biodiversity loss. This study explores the intricate link between human trampling, plant density, and the morphological and reproductive characteristics of *Globularia alypum* L., a plant with a peripheral population in Sardinia restricted to a natural area within an urban context. The study examined trampling effects on 75 plots along a frequently used path crossing the plant’s core population. Similar environmental conditions were assumed, with differences attributed to human trampling intensity and plant density, and morphological and reproductive traits were measured within each plot. Our results showed that human trampling caused differences in the morphological traits of plants, whereas, in contrast, reproductive traits are less vulnerable to human trampling than morphological ones. As a result, trampled areas may experience decreased plant recruitment, which can have long-term implications for plant population dynamics. Understanding the relationship between trampling effects and the sensitivity of peripheral plant populations is crucial for effective conservation and management strategies.

## 1. Introduction

Natural areas, especially in an urban context, are important resources providing opportunities for people to engage with nature [1,2] and, lately, interest in nature-based recreation and the appreciation of natural areas has increased worldwide [3,4]. These areas, besides being popular tourism destinations, provide a wide range of ecosystem services, such as protecting soils and conserving biodiversity, and could contain important archaeological and cultural sites [5,6,7,8,9,10,11]. Furthermore, these green areas are valuable habitats for a wide variety of plant and animal species that depend on natural urban areas as crucial habitats. An increasing number of scientific studies have considered many sites within cities as places that have the ability or the potential to provide refugia for numerous native species. Therefore, several species, due to the abundance of unique, unusual, and intricate habitats found in urban landscapes, can find refuge in these habitats, which are very different from those that surround cities [12]. The biodiversity found within natural areas has become one of the primary attractions for prospective visitors, who find themselves enjoying the beauty that biodiversity provides and the consequent sense of place that derives from it [7], raising public awareness of the need to conserve it [13,14,15,16,17]. The importance of enjoying these benefits is such that it has been linked to physical and mental health benefits, particularly for communities living in large urban cities [18,19,20]. As a result, the sustainable coexistence of both nature conservation and tourism is one of the aims of sustainable tourism, namely ecotourism [21].

Nevertheless, the growing demand for access to these areas and the rapid expansion of recreational activities may endanger the environmental characteristics sought by visitors, causing biodiversity loss, soil erosion, and pollution [4,22,23,24,25,26,27]. In recent decades, the increased use of natural and wild areas for recreational activities has been identified as a threat to these natural ecosystems [4,28,29,30,31,32]. Particularly for plants, human recreational activities such as walking, hiking, and biking could alter the density of individuals in a population and the morphological and physiological features of species by causing direct physical loss or damage to them [33,34]. Human trampling is one of the most common and visible forms of disturbance to plants and can occur when recreational users leave an established trail to take a photograph, investigate a flower, or create an informal trail for their purpose [15,22,26]. However, there is a species-specific variability in susceptibility to human trampling among plants, which varies, sometimes markedly. Indeed, the distribution of visitors and their recreational activities have different effects on plant populations [35,36,37] based on the threat level of the populations [38,39,40,41]. Generally, fast-growing plants show high resistance to human trampling [33,35,42,43,44] or seem to be tolerant of, or even benefit from, visitor pressure, such as human trampling [45,46]; on the other hand, plants growing in peripheral populations, isolated from the main home range populations, and threatened endemic plants can more markedly suffer the threat of disturbance [36,38,40,47,48]. Among the other consequences, human trampling represents an important threat to single plants or plant populations because it could determine a reduction in plant density, an alteration in morphological parameters, and a consistent reduction in reproductive traits [38,40,49], resulting in its posing a serious threat to the persistence of the populations. These negative impacts might be propagated to higher levels, such as weakening ecosystem stability and functioning due to loss of vegetation cover, changes in species composition and diversity, and an increased risk of invasive species [23,26,31,50,51,52,53]. Understanding the relationship between the effects of human trampling and the sensitivity of a plant in peripheral populations is an important but complex issue because those populations, already vulnerable due to genetic factors and edge effects, could be more sensitive to human trampling disturbances [36,37]. In the Mediterranean Basin, one noteworthy characteristic of many plant species is the occurrence of small, peripherally isolated populations within natural areas, often near urban areas [36,54,55,56].

This paper focuses on the impact of human trampling on the only small population of *Globularia alypum* L. (Plantaginaceae) in Sardinia. On the island, it thrives exclusively in a natural area situated within an urban context, specifically the “Capo Sant’Elia” promontory in the municipality of Cagliari. So, *G. alypum*, with its marginal, disjunct, and isolated population, exemplifies a phytogeographical entity of significant conservation concern. Its presence signifies the intricate interplay of environmental, geographical, and historical factors that have shaped its geographical distribution. Therefore, its conservation is of critical importance, not only because it contributes to the richness of biological diversity in the entire area but also because it can play pivotal roles within the ecosystems it inhabits. This area is frequented by residents engaging in various sports and leisure activities like mountain biking, hiking, and free climbing. Over the past five decades, rapid urban growth has significantly impacted these locations. However, despite these challenges, the hills still retain a considerable level of naturalness, especially in the urban zone, where natural vegetation persists, serving as unique reservoirs of biodiversity. In the framework of our long-term studies in this area focused on plants and habitats of conservation interest, we observed an intense touristic traffic along the paths that affects plant species and communities.

Consequently, the primary objectives of this study were twofold: (1) to explore how human trampling affects the population density and the morphological and reproductive characteristics of phytogeographically interesting species *Globularia alypum*; (2) to understand which conservation implications are recommended to conserve the plant population in this natural area.

## 2. Results

A total of 91 individuals of *Globularia alypum* were found and measured within the plots, of which 47.2% were both in the Intermediate and the Extreme plots, while only 5.6% were in the Central plots. 

**Figure 1:**
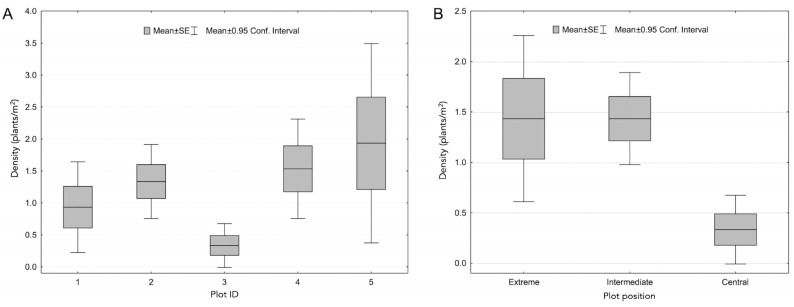
Plant density at Plot ID (**A**) and Plot position (**B**) level.

**Figure 2:**
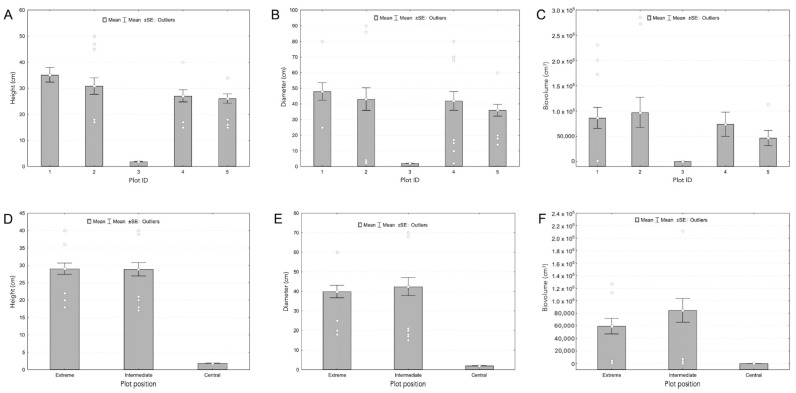
Morphological plant traits at Plot ID (**A**–**C**) and Plot position (**D**–**F**) level.

The plant density markedly differed among plots, depending on position; in particular, in Central plots, the lowest mean values (±SD) were recorded (0.33 ± 0.61 plants/m^2^) while similar and higher mean values than in Central plots were recorded in Intermediate and Extreme plots (1.43 ± 1.23 and 1.43 ± 2.20 plants/m^2^, respectively). The Kruskal–Wallis One-Way Analysis of Variance on Ranks test revealed a statistically significant difference among Central and Intermediate plots (*p* < 0.05), while no difference was found among Central and Extreme plots (*p* > 0.05); no significant differences were found among Intermediate and Extreme plots (*p* > 0.05; Table 1; Figure 1 and Figure 2).

Human trampling disturbance caused pronounced reductions among groups of plots in morphological traits such as plant height and plant size (Table 1). Specifically, the mean values of plant height are very different for plants growing in the Central, Intermediate, and Extreme plots (1.88 ± 0.25, 28.86 ± 12.71, and 29.04 ± 10.74 cm, respectively). The Kruskal–Wallis One-Way Analysis of Variance on Ranks test revealed a statistically significant difference both between Central and Extreme plots and Central and Intermediate plots (*p* < 0.05); no differences were found among Intermediate and Extreme plots (*p* > 0.05; Table 1; Figure 1 and Figure 2). Analogously, the mean values of plant diameter showed low values for Central plots and similar mean values in the Intermediate and Extreme plots (2.02 ± 0.14, 42.4 ± 30.04, and 39.90 ± 20.93 cm, respectively). Also, for this parameter, the Kruskal-Wallis One-Way Analysis of Variance on Ranks test revealed a statistically significant difference between Central and Extreme plots and Central and Intermediate plots (*p* < 0.05); no differences were found among Intermediate and Extreme plots (*p* > 0.05; Table 1; Figure 1 and Figure 2).

Plant biovolume revealed the same trend of density (6.01 ± 0.91, 84,693.06 ± 123,741.9, and 59,472.74 ± 81,497.3 cm^3^ for Central, Intermediate, and Extreme plots, respectively) and, based on the Kruskal–Wallis One-Way Analysis of Variance on Ranks test, showed a statistically significant difference between Central and Intermediate plots and Central and Extreme plots (*p* < 0.05). No differences were found among Intermediate and Extreme plots (*p* > 0.05; Table 1; Figure 1 and Figure 2).

Only two reproductive plants were found in the Central plots, in contrast to what was observed in Intermediate and Extreme plots (86% reproductive plants); however, the Kruskal–Wallis One-Way Analysis of Variance on Ranks test did not show a statistically significant difference among plots (*p* > 0.05; Table 1; Figure 1 and Figure 2).

## 3. Discussion

Natural areas are invaluable ecosystems that support diverse plant and animal species, providing crucial ecological services. However, the plant populations found in these fragile places may suffer because of the rising popularity of outdoor leisure and the ensuing rise in human traffic. This has been demonstrated by extensive research efforts dedicated to studying these phenomena and investigating the impacts of outdoor recreation on delicate ecosystems [23,35,50]. These studies reported that human recreational activities induce mechanical disturbances within natural habitats, adversely affecting vegetation by altering coverage, species composition, diversity, and plant height, and exacerbating the risk of invasive species or weed proliferation [15,23,51,53,57]. Other studies concentrated on the compaction of soil resulting from recreational activities, which diminishes soil water retention capacity, potentially escalating erosion rates and water runoff. Therefore, this can lead to severe disturbances, altering the composition and functioning of peripheral plant communities [36,37].

Our research, focusing on how human trampling affects *G. alypum*, an interesting phytogeographic plant with a peripheral population, demonstrated that continuous human trampling on paths that cross the site where the species grows can reduce the plant’s density, alter its morphological traits, and, consequently, progressively damage the persistence capacity of this population. This phenomenon is a common issue in many natural areas, especially those with high visitor foot traffic, such as popular hiking trails, parks, and natural reserves [5].

Human trampling causes direct and indirect disturbances to *Globularia alypum* that are not uniformly distributed in the plant population. Our study confirmed a marked concentration of human trampling within the central region of the path, with more frequent and intense trampling compared to the edges. This non-uniform distribution can lead to different plant responses in these two areas. The statistically significant differences between the Central plots and the Intermediate ones demonstrate that human trampling can have pronounced effects, such as the physical damage inflicted on plants. As individuals walk along the central path, plants in their way often get crushed or uprooted, leading to a decline in plant density in this area. This damage can be particularly severe, impacting the survival of individual plants. In addition to direct plant damage, human trampling compacts the soil, affecting the ability of plant roots to access essential resources. The central path, subjected to intense human trampling, experiences more pronounced soil compaction, which reduces the density of plant populations [9,31,38,58,59,60]. Meanwhile, adjacent areas (Intermediate plots) that are less affected by human trampling and experience a more buffered impact provide more favourable conditions for the survival of plants and the recruitment of new individuals. Conversely, no significant difference in mean plant density was found between the Central and Extreme plots, where the impact of human trampling should be irrelevant or absent; the explanation for this pattern lies in the fact that *G. alypum* generally grows in open scrublands while, at the edges of the path, micro-forest formations develop, which reduce the ecologically suitable spaces for our species to the point of determining their disappearance when the formation becomes closed. 

The marked, statistically significant, differences in growth traits between the Central and peripheral (Intermediate and Extreme) zones of the path confirm, as demonstrated by [61], that these traits are sensitive to human trampling, which may lead to differential impacts on population persistence. In fact, we found that human trampling disturbance reduced the overall plant size, particularly the height and the maximum diameter, and, consequently, the plant biovolume, as a probable consequence of direct physical damage, including the breakage of stems, branches, and leaves. In our specific case, the reduced height and diameter can cause this species to have a higher sensitivity to human trampling than other perennials [37,47,49] or small herbaceous or annual plants [38,40]. 

Similarly to what was demonstrated by [61], our study showed that the morphological traits of *G. alypum* are vulnerable to human trampling, while the reproductive ability appears not to be significantly affected. In fact, contrary to what was expected, our results provided no support for the hypothesis that human trampling causes differences in the reproductive capacity of *G. alypum* regardless of individual size. This resilience to human trampling could be attributed to various reproductive strategies but, above all, to the reproductive timing: *Globularia alypum*, in fact, experiences flowering and fruiting in autumn and early spring, seasons in which the frequency of human trampling is lower due to weather conditions.

Plants such as *G. alypum*, which has phytogeographical importance in Sardinia, are often restricted to specific areas. In this particular instance, the urban setting can be seen as a special refugium that offers vital habitats and new opportunities for species to survive in an environment marked by disturbance and stress [12,62,63,64]. Isolated populations have unique biological and ecological requirements, making them of interest in ecology, evolutionary biology, and genetics. In urban areas, common nature-based activities like hiking can significantly impact these populations, affecting their unique conditions [37,65,66,67]. The loss of these unique populations could considerably affect community dynamics and reduce the overall biodiversity of urban natural areas.

## 4. Materials and Methods

### 4.1. Study Species and Study Area

**Figure 3:**
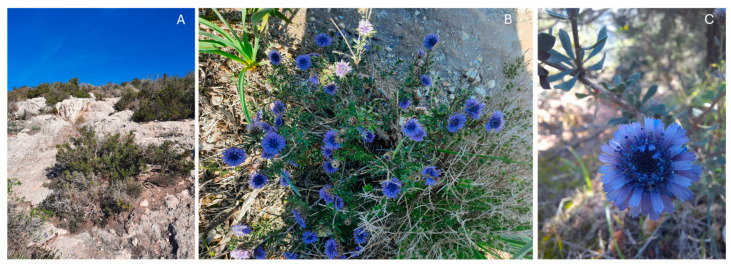
(**A**): representative habitat where the species grows; (**B**): *Globularia alypum* individual; (**C**): *Globularia alypum* details.

*Globularia alypum* L. (Plantaginaceae) is a nanophanerophyte shrub reaching 0.3–1 m in height (Figure 3). It has perennial sclerophyllous leaves in an alternated and scattered arrangement. Flowers are hermaphrodite, tiny, and gathered in a hemispherical flower head of 2–3 cm; fruit consists of a nucula, containing 1–2 mm sized seeds [68]. However, there is a lack of available information about the reproduction and seed dispersion of *G. alypum*. Flowering begins in the autumn months, usually in October–November, and lasts until March–May [68].

*Globularia alypum* is a heliophilous and xerophilous plant species growing in calcareous soils and is typical of termophilous shrublands (Figure 3); it is mostly distributed in the coastal areas of the eastern Mediterranean Basin (Crete, Cyprus, Greece, Israel, Italy, Jordan, Lebanon, Libya, Palestine, and Turkey [69]. In Italy, *G. alypum* can be found in Sicily, Sardinia, the islands of Elba, Egadi, and Lampedusa, from sea level to 600 m a.s.l. in the western Liguria and Tuscany regions [68].

The westernmost population, which is extremely small and completely isolated by the nearby populations, occurs in an urban natural area in Sardinia (Capo S. Elia promontory; Figure 3), where Moris originally noted it in 1827 [70]. The Capo Sant’Elia promontory is an area of c. 200 ha and a maximum height of 136 m a.s.l., surrounded by the city of Cagliari (39°11′ latitude north, 9°10′ east longitude), the largest city in Sardinia.

The bioclimatic analysis of the study area was carried out using thermo-pluviometric data recorded by the nearest weather station (Cagliari), which showed a mean annual temperature of 17.9 °C and an annual precipitation of 394.0 mm [71]. According to the bioclimatic classification proposed by [72], this area is classified as Mediterranean Pluviseasonal Oceanic (MPO), with an upper thermomediterranean thermotype, and an upper dry ombrotype [71]. The Capo S. Elia promontory, along with the calcareous hills rising in the southern part of the Campidano plain, is the only limestone emergency in south-eastern Sardinia, retaining high levels of biodiversity [73]. It is distinguished by its high floristic diversity and by the presence of endemic flora and other plant species of biogeographical interest. Among these, *Sarcopoterium spinosum* (L.) Spach and *Globularia alypum* L. are two highly significant phytogeographical plant species that, in Sardinia, are exclusively present in this promontory [74]. 

### 4.2. Sampling

**Figure 4:**
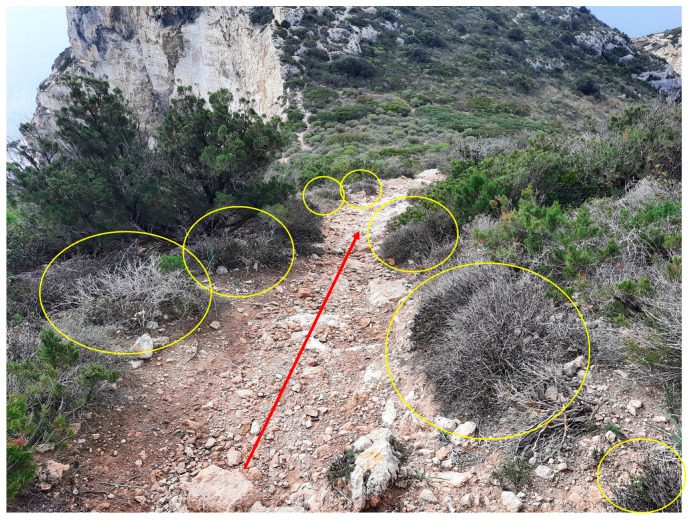
Representative section of the path where nearby growing *Globularia alypum* L. Red arrow shows the trampled area of the path.

In 2021, to investigate the effects of human trampling on *G. alypum*, the most frequented path that crosses the core of the population was selected (Figure 4). Over the year, the study area was examined through numerous field trips that allowed for direct observation of the ongoing presence of visitors, which caused the trampling of plants and/or the breaking of branches.

**Figure 5:**
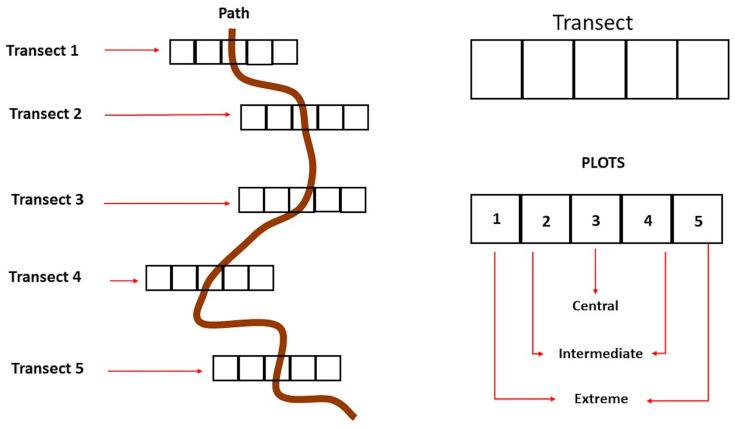
Exemplary diagram of the layout of the transepts and plots in the experimental design, adopted to analyse the effect of human trampling on the *Globularia alypum* L. population present at Capo Sant’Elia Promontory (Cagliari, Sardinia).

To test our hypothesis that touristic trampling has a negative effect on plants, an experimental plan based on previously established protocols, e.g., [31,38,75], and appropriately modified based on the characteristics of our species, was developed. Specifically, along the path, a stretch of 100 m was selected where perpendicular transects were randomly positioned. A total of 15 transects, each consisting of five adjacent plots (hereafter P1, P2, P3, P4, and P5) of 1 × 1 m, were randomly individuated. Overall, 75 plots were identified and included in this study (Figure 5). The first plot was located at the centre of the path (P3, positioned to coincide with the area most subject to human trampling; see Figure 5); the second and third plots (P2 and P4) were positioned at a distance of 0.50 m from the centre of the path to the left and the right of the path (furthest from the path and less subject to human trampling; Figure 5); the last two (P1 and P5) were 1.5 m away from the centre of the path, to the left and the right of the path (Figure 5).

The selected 15 transects presenting similar environmental conditions (substrate, aspect, and slope). We therefore assumed that any differences between plots were a result of the intensity of human trampling. Within the plots, all individuals of *G. alypum* were counted, and for each one, both morphological (height and diameter) and reproductive traits (flowering and fruiting plants) were also counted.

### 4.3. Statistical Analysis

To estimate the plant size, the volume of each plant (*V*, expressed in cm^3^), understood as the smallest solid that contains the entire individual, was calculated according to the following formula:

(*V* = *π* × *r*^2^ × ℎ)

where *r* indicates the “radius”, calculated as half of the major diameter, and *h* is the “plant height” (cm) of a single plant.

To analyse the human trampling effects related to the position of the plot on the plant density, plant morphological traits, and the reproductive capacity of the plant, the plots considered within the transects were grouped as follows: Central plots (P3), Intermediate plots (P2 and P4), and Extreme plots (P1 and P5), following a gradient of progressive distancing from the path, and therefore from the source of the human trampling disturbance.

To verify statistically significant differences in density, plant size, and morphological and reproductive parameters among groups of plots at different distances from the centre of the path, the Kruskal–Wallis nonparametric test was applied, followed by pairwise multiple comparison tests (Kruskal–Wallis One-Way Analysis of Variance on Ranks test). All analyses were performed using Statistica 8.0 software (Statsoft, Tulsa, OK, USA).

## 5. Conclusions

Human trampling linked to recreational activities is a common disturbance to animal and plant species caused by human activities, particularly in recreational areas and natural habitats. In addition to having an influence on individual plants, this anthropogenic pressure can have a major effect on plant populations, especially those found in peripheral or edge locations. Plants with a peripheral population, such as *G. alypum*, should be considered of high interest, with a need for urgent conservation measures.

Because of the distinctive ecological features that make peripheral populations more susceptible to disturbances, it is essential for conservation and management methods to comprehend the relationship between the sensitivity of peripheral plant populations and the consequences of human trampling.

In particular, greater emphasis should be placed on minimising the range of negative impacts, including unsustainable tourism and recreation use [22,38]. The main international strategies for plant conservation emphasise the importance of protecting populations at the edge of their distribution range, like *G. alypum* in Capo Sant’Elia promontory in Sardinia. Several strategies can be used to lessen the negative effects of human trampling on marginal plant populations in natural regions. By designating certain trails and walkways, foot traffic may be concentrated, and the spread of human trampling in vulnerable regions can be inhibited. Additionally, the harmful effects of human trampling can be minimised by teaching visitors to natural places about the ecological worth of these species and encouraging appropriate outdoor activities.

Finally, long-term monitoring programmes are crucial to address future threats such as climate change and biological invasions. These programmes should track changes in species conservation status, vegetation, and human-induced threats.

## Figures and Tables

**Table 1:** Results of the Kruskal–Wallis One-Way Analysis of Variance on Ranks test regarding the plant density, and morphological and reproductive traits of *Globularia alypum* at different distances from the centre of the path. Significant values are in bold.

Parameter	Multiple Comparisons z’ Values; Kruskal–Wallis Test		Plots		
	H_(2, N = 75)_ = 9.5003 *p* = 0.0087		Extreme	Intermediate	Central
Plant density		Extreme		1.5164	1.6420
		Intermediate			**2.8801**
		Central			
Plant height	H_(2, N = 91)_ = 13.6637	Extreme		0.3368	**3.6463**
	*p* = 0.0011	Intermediate			**3.4926**
		Central			
Plant diameter	H_(2, N = 91)_ = 2.7551	Extreme		0.1143	**3.5192**
	*p* > 0.05	Intermediate			**3.4670**
		Central			
Plant biovolume	H_(2, N = 91)_ = 13.1038	Extreme		0.1939	**3.5628**
	*p* = 0.0014	Intermediate			**3.4743**
		Central			
Reproductive status	H_(2, N = 91)_ = 10.2051	Extreme		1.1145	1.9331
	*p* = 0.0061	Intermediate			1.4244
		Central			

## Data Availability

Detailed data are available from the corresponding author upon request. The data are not publicly available because they are currently the topic of additional research.
